# Long-term tracking of budding yeast cells in brightfield microscopy: CellStar and the Evaluation Platform

**DOI:** 10.1098/rsif.2016.0705

**Published:** 2017-02

**Authors:** Cristian Versari, Szymon Stoma, Kirill Batmanov, Artémis Llamosi, Filip Mroz, Adam Kaczmarek, Matt Deyell, Cédric Lhoussaine, Pascal Hersen, Gregory Batt

**Affiliations:** 1 BioComputing, CRIStAL, Université Lille 1, Lille, France; 2 Scientific Center for Optical and Electron Microscopy (ScopeM), ETH Zurich, Zurich, Switzerland; 3 Laboratoire Matières et Systèmes Complexes, UMR7057, CNRS and Université Paris Diderot, Paris, France; 4 Inria and Université Paris-Saclay, Palaiseau, France; 5 Institute of Computer Science, University of Wroclaw, Wroclaw, Poland

**Keywords:** image analysis, segmentation and tracking, parameter learning, imaging benchmark

## Abstract

With the continuous expansion of single cell biology, the observation of the behaviour of individual cells over extended durations and with high accuracy has become a problem of central importance. Surprisingly, even for yeast cells that have relatively regular shapes, no solution has been proposed that reaches the high quality required for long-term experiments for segmentation and tracking (S&T) based on brightfield images. Here, we present *CellStar*, a tool chain designed to achieve good performance in long-term experiments. The key features are the use of a new variant of parametrized active rays for segmentation, a neighbourhood-preserving criterion for tracking, and the use of an iterative approach that incrementally improves S&T quality. A graphical user interface enables manual corrections of S&T errors and their use for the automated correction of other, related errors and for parameter learning. We created a benchmark dataset with manually analysed images and compared *CellStar* with six other tools, showing its high performance, notably in long-term tracking. As a community effort, we set up a website, the Yeast Image Toolkit, with the benchmark and the *Evaluation Platform* to gather this and additional information provided by others.

## 1. Introduction

Observing cellular processes at the single cell level is often necessary to understand how cells respond to endogenous and environmental changes. Used in combination with fluorescence reporter techniques, flow cytometry and time-lapse microscopy are arguably the two most widely employed quantitative single-cell observation approaches. The former provides great statistical details on the diversity of the studied cell population, whereas the latter provides longitudinal information on single cells: individual cells can be tracked in time. This is a decisive advantage to investigate a number of important biological problems, including chronological ageing, epigenetic heritability and dynamic features such as the cell cycle and circadian oscillations in non-synchronized cell populations. One can take advantage of microfluidic microchemostat that, unlike liquid bulk culture, enables long-term observations of cells growing as a monolayer. Moreover, microfluidics can be used to create time-varying environments. Both aspects are of increasing importance to obtain a quantitative understanding of cellular processes at the single cell level.

**Figure 1.**
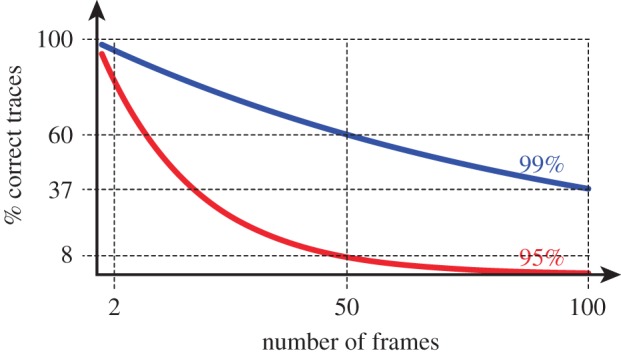
Precision decay in long-term tracking. Tracking quality decreases exponentially with the increase in the number of frames. Assuming that the probability *ρ* of a correct cell assignment (i.e. a cell in one frame corresponds to the same cell in the previous frame) is constant in time, then the probability of the trace being correct (i.e. to describe always the same cell) is 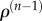, with *n* being the length of the trace (i.e. the number of frames). If the probability of correctly mapping a cell across two consecutive frames is 99%, then the probability that a trace spanning 100 frames is correct is only 37%.

However, the capability to extract *single cell traces* from microscopy images in a fully automated manner is a necessary prerequisite to obtain conclusions that are valid and biologically relevant in long-lasting experiments. Incorrect assignments (e.g. two cells exchanged at some time point) can possibly hide interesting features or worse, create spurious information. Although such incorrect assignments are expected to be relatively rare at each time point, a simple analysis shows that the number of correct traces decreases rapidly with the duration of the experiment (figure 1).

In the following, we focus on the analysis of microscopy images of the budding yeast *Saccharomyces cerevisiae* observed in brightfield. While fluorescent markers can be used to tag cellular compartments and significantly ease the image analysis problem, brightfield imaging circumvents the need for genetically engineering cells and avoids dedicating one precious fluorescent channel to find cells' contours, together with possible phototoxicity effects coming from fluorescence imaging.

The extraction of cell traces is usually separated into two distinct tasks: segmentation and tracking. The aim of segmentation is the detection of the areas (technically called segments) occupied by each cell in each image. Tracking maps each segment in one image to one (or no) segment in the following image, so that the history of each cell is reconstructed over the entire duration of the experiment. Segmentation and tracking of yeast cells in microscopy images are widely studied problems [1–14]. Usually, segmentation is obtained through a combination of a few basic image operations: intensity thresholding, filtering and other morphological operations [15]. Other classical methods use region accumulation approaches such as Voronoi-based methods [16], the watershed transform [17] or deformable-model approaches such as active contours [18,19]. Methods and tools for cell segmentation and tracking have been described in reviews such as [15,20,21].

Nevertheless, yeast single cell segmentation and tracking are still frequently a technical bottleneck, for example as a consequence of the difficulties in the tuning of image-processing parameters, the meaning of which is mostly obscure for the average user. Most of the time researchers resort to home-made solutions based on semi-automated tracking systems. Such methods generally fail to robustly recover cell trajectories, or at best are tailored for a very specific experimental system, usually relying on additional fluorescent markers or constrained microfluidic geometry forming cell traps.

In this paper, we present *CellStar*, a tool chain for the analysis of videomicroscopy data in which all the steps have been designed to meet the quality requirements needed for the analysis of long-term experiments using budding yeast cells. This has been achieved by the application of iterative algorithms that incrementally gather information from the image in order to make cell segmentation and tracking *robust* with respect to the most common image analysis errors. In particular, for segmentation, we use a new variant of *active rays*, which exploits information regarding the interior of contours. Active rays, also called polar active contours, are a computationally efficient framework for the identification of object outlines in which the contour extraction problem is defined as an energy minimization problem and contours belong to a family of parametric curves [22]. For tracking, we use a multi-criteria optimization algorithm. It notably includes the penalization of relative displacements between neighbours proposed by Delgado-Gonzalo *et al.* [23], which provides robustness to collective cell movements. The high-quality results obtained by automatic image processing can be further improved manually thanks to *CellStar*'s graphical user interface (GUI). Manual corrections are also exploited for automatic parameter learning, which relieves the user of understanding the trickiest parameters of the algorithms.

Furthermore, we compare *CellStar* with other segmentation and tracking tools. We developed a manually curated set of yeast microscopy images to be used as a benchmark. Indeed, no consensus has emerged yet on the best-performing tool, and no systematic analysis of their performance has been proposed for long-term videomicroscopy data. We thus selected images that reflect a diversity of situations encountered in typical experiments. We compared *CellStar* with six software solutions dedicated to yeast cell segmentation and tracking in brightfield microscopy, namely CellID [1], CellTracer [6], CellSerpent [7], CellX [12], Tracker [24] and the intensity-based segmentation-overlap-based tracking (IBSOBT) pipeline for CellProfiler [25] (see electronic supplementary material, table S2). These tools have been selected for their representativity, together with the availability and usability of their implementation. Other dedicated tools or image analysis platforms could have been considered [2–5,8,10,11,13,14,26].

In our comparative analysis, we found that *CellStar* outperforms the other tools we tested. Naturally, these results should be interpreted with care because they have been obtained on data produced in our laboratory and with the best parametrization we could find for each tool, which might not be the optimal one. Therefore, this study does not aim to provide definitive conclusions but rather to initiate a community effort to compare tools on the same data. To this end, we additionally set up a companion website, *Evaluation Platform*, enabling segmentation and tracking results to be compared and updated when new benchmarks or new tools become available. *CellStar*, the *Evaluation Platform* and the benchmark dataset are freely available on the website.

## 2. Results

### 2.1. Segmentation

Segmentation is often a key phase of image processing, during which each image is processed independently with the aim of identifying the boundaries of every cell. In order to meet the precision requirements needed in long-term experiments, we designed a segmentation pipeline based on a new variant of polar active contours [22]. The pipeline has been conceived to provide high segmentation quality in the presence of several problematic conditions typical of mono-layer cultures of growing yeast cells, including images fully crowded with packed cells and wide range of cell sizes. Here we simply describe the main steps of the approach, highlighting the specific features that all together enable effective long-term tracking. A detailed description of the pipeline including the mathematical formalization of active contours and the specification of the main algorithm in pseudo-code is provided in electronic supplementary material.

#### 2.1.1. Active contours with interior

**Figure 2.**
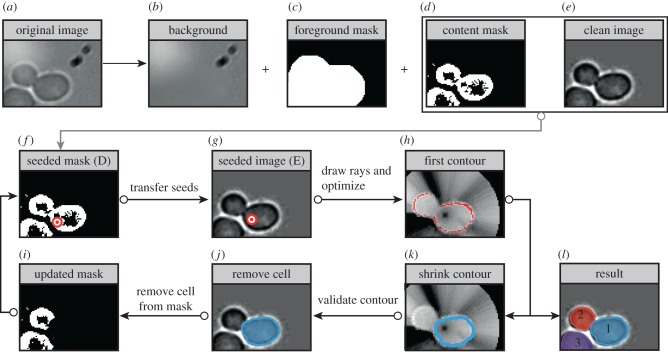
Segmentation pipeline: preprocessing, seeding and contour deformation. The background (*b*) is computed and subtracted from the original image (*a*) to give the clean image (*e*). Preprocessing steps also include the computation of the foreground (*c*), cell border (not shown) and cell content (*d*) masks. These masks are used to place initial seeds, represented by red/white dots (*g*). Starting from each seed, the initial shape of the snake (*h*) is computed by drawing concentric rays, computing an approximated *E*_snake_ and selecting its minimum along each ray; the initial shape of the snake is modified according to the given regularity constraints to obtain the final contour (*k*). After filtering (figure 3), selected contours (*j*) are removed from the border and content masks, as shown in (*i*) so as to ease the discovery of new cells. The final result is shown in (*l*).

Active contours (or snakes [18]) are a well-known framework for the identification of objects outlines. Informally speaking, active contours are deformable curves that are pulled or pushed towards the boundaries of the objects to be identified. In the case of roundish cells like yeast, closed active contours are usually exploited for segmentation, resembling a sort of balloon in which inflation or deflation is applied to match the contour of a cell [2,7]. Snakes are deformed to minimize a weighted energy, *E*_snake_, whose traditional definition has been adapted as follows


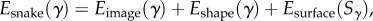
where *γ* is the curve corresponding to the snake and *S*_γ_ its interior. *E*_image_ represents an energy term related to the intensity, the intensity gradient and terminations of the image underlying contour (*γ*). It is the line integral
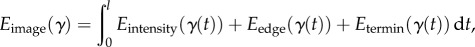
where *l* is the length of *γ*. Here, we exploit the fact that (i) cells appear darker than the background, (ii) cell borders are characterized by a high-intensity gradient (effect of transition from darker inside to brighter outside), and (iii) in dense settings, intercell space is significantly brighter (figure 2*a*). More details are provided in the electronic supplementary material. *E*_shape_, also often called *E*_internal_, depends on the geometrical properties of the snake. Note that in comparison with the traditional definition, the definition of *E*_shape_ has been modified to take into account the area of the interior of the snake:
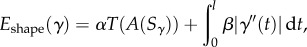
where *A*(*S*_γ_) represents the area of the interior of *γ*. The first term accounts for the extent of the snake and the second term measures the regularity of the contour. 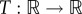 is an arbitrary (continuous) function that allows the introduction of a bias in the energy term depending on the area of the snake, for example to favour the detection of cells with some expected average size.

We observed that including an additional term incorporating properties of not only cell contour but also the cell interior leads to significant improvements in segmentation results. With 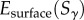, we propose a new energy function for closed active contours that critically differs from the usual definition, thanks to the presence of terms related to contour *interiors*:
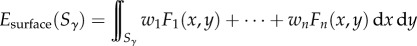
with 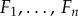 being user-defined image filters weighted by 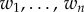. By default, we use as filters only the cell content and cell border images. *E*_surface_ allows us to exploit information about the content of snakes, which can increase the quality of segmentation when contours are not continuous and sharp, as it happens in images of packed cell clusters. In particular, it allows us to substantially extend the range of cell sizes that can be detected without succumbing to under- and over-segmentation. Note that *E*_shape_ was already deviating from classical definitions of the energy for active contours that usually exclusively use properties of the pixels lying on the contour itself. Consequently, optimizations need to be done on two-dimensional structures rather than on one-dimensional structures, possibly leading to significantly larger computational costs. A complete formalization of the above energy terms is provided in the electronic supplementary material.

#### 2.1.2. *CellStar* segmentation pipeline

Minimization of the energy of active contours is a key element to segment each single cell in the image. However, it is only a part of the *CellStar* segmentation pipeline consisting of a few independent steps. First, original images are preprocessed to create helper images. Initial contours (called seeds) are then placed on the image. Next, the shape of each contour is modified independently by minimizing 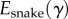, until it matches the outline of a cell. The procedure of seeding, contour shape modification and filtering is repeated until the area with cells is completely covered with contours or a given number of iterations is reached.

From the computational perspective, contour deformation is an expensive part of the algorithm. To lower the computational complexity, we use polar active contours [22]. The class of shapes allowed for polar active contours corresponds in practice to star-shaped polygons: a star-shaped polygon is such that there exists (at least) one point in its interior (referred to as the centre of the polygon) from which the entire polygon is directly *visible*. Polar active contours provide numerous advantages for the segmentation of roundish cells like yeast: a more efficient energy minimization process, obtained by the reduction of the freedom of contour control points from two dimensions to one and the absence of contour self-crossing by design. In the remainder of the section, the main concepts of the pipeline are briefly described. We refer the reader to the electronic supplementary material for a more formal description of the pipeline.

*Preprocessing.* The aim is to compute a set of intermediate images representing all the features exploited later on in the proper segmentation phase, including the filters 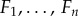 previously mentioned. They are obtained by applying classic filters for the attenuation of noise and illumination artefacts, edge detection, as well as several custom filters for the computation of binary masks necessary for the efficient placement of initial contours and their subsequent deformation (e.g. background image, foreground mask, cell content and cell border masks). Examples of these images are shown in figure 2*b*–*d*.

*Seeding.* Seeding seeks to find the centres of future contours (figure 2*f*–*g*). Tentative initial seed positions are obtained by looking for brightness minima after having applied smoothing filters to the cell border image. This seeding strategy is similar to that of [7]. Several other strategies are then applied using the cell content image, previously discovered segments and randomization (see the electronic supplementary material for details). Ideally, our procedure places exactly one seed inside every cell. In practice, some cells get more than one seed, whereas others get none. In the case of cells with multiple associated seeds, it is usually enough to choose the most ‘promising’ final contour (see the ranking and filtering phase below). Cells with no associated seeds represent instead a serious problem because they end up missing in the segmentation. Instead of overfilling the image with many seeds at once, the *CellStar* pipeline performs several seeding iterations where new seeds are placed in those areas of the image where cells are expected but have not yet been found. This is achieved by excluding the areas already covered by contours from some of the images computed in the preprocessing phase, in particular the cell content and cell border images (figure 2*i*–*j*). This is an important step of the strategy. In this way, the *CellStar* pipeline minimizes the number of missing cells efficiently from a computational perspective.

*Contour deformation.* This is the most computationally intensive phase of the segmentation process. To minimize the computational cost, the centre of the related star-shaped polygon is not modified during contour deformation. Additionally, the number of vertexes of the polygon are fixed and their movement is constrained along straight lines originating from the centre (polar active contours are also called active rays; figure 2*h*). We implemented ad hoc heuristics for the minimization of contour energy, based on a number of approximations of the energy function, to provide acceptable trade-offs between segmentation quality and computation time even in the most demanding situations, such as real-time image analysis ([24]; figure 2*h*,*k* and electronic supplementary material).

**Figure 3.**
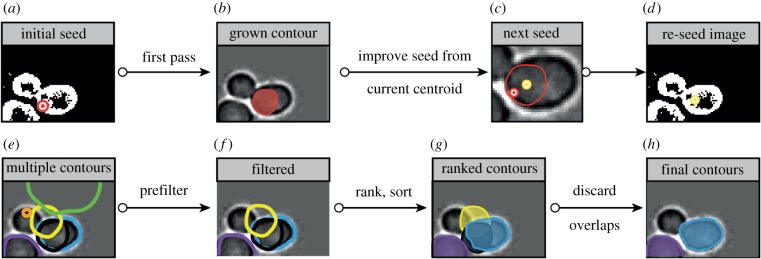
Contour seeding and ranking. (*a*–*d*) Improving contour detection by seed relocation. In (*b*), a first contour is found starting from an initial, badly located seed (*a*). In a second step (*c*), the centroid of the detected contour becomes a new seed (*d*), as it usually falls closer to the real centroid of the cell and allows improved contour detection. (*e*–*h*) Ranking and filtering. All the ‘bad’ contours (*e*) (too big, too small, not lying in the foreground mask, etc.) are discarded. The remaining ones (*f*) are ranked and successively placed on the image (*g*). Contours that overlap with previously placed contours are discarded. Another round of segmentation may be started.

*Ranking and filtering.* Contour ranking and filtering is the last phase of segmentation, where snakes are kept or discarded according to several criteria. Snakes are ranked according to *E*_snake_, then those overlapping with other, lower-energy snakes are discarded according to the hypothesis that they correspond to a worse detection of the same cell, as shown in figure 3. This procedure is similar to what is done for CellSerpent [7], however, in our pipeline the ranking and filtering phase becomes an essential part of the search of optimal contours, thanks to the seeding strategies previously described and further detailed in the electronic supplementary material.

### 2.2. Tracking

During segmentation of a specific frame, each cell is assigned with a numerical label called a (*cell*) *detection* number. Tracking is then defined as the task of associating each detection in each frame with a unique cell identifier. Cell movement is the main obstacle that has to be overcome during tracking. Cells move across, appear in and disappear from the field of view. First, we present an approach to relate cells in two successive frames assuming that cells move independently of one another. While this assumption is not always true (clumped cells tend to move together), it greatly simplifies the algorithm. Second, we relax this assumption and propose an improvement of the approach.

#### 2.2.1. Independent motion assumption

We consider that detections of cells in frame *i* depend only on detections in frame *i* −1. Therefore, we can introduce a frame-to-frame assignment matrix 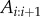 that specifies appearing and disappearing cells as well as the presence of the same cell in frames *i* and *i* + 1. The problem is thus reduced to finding the best frame-to-frame assignment matrix.

To do so, we introduce a cost matrix 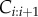 that specifies for each cell detection number the cost of assuming that it corresponds to an appearing or disappearing cell, and for each pair of cell detection numbers, the cost of assuming that they correspond to the same cell in frames *i* and *i* + 1. The cost of missing cells is based on the distance of cell detections from frame borders (cells near borders tend to appear and disappear more frequently). The cost of associating two detections with a single cell depends on how similar they are, that is their relative shapes and distance between the two-dimensional coordinates of the frames.

As shown in the electronic supplementary material, the best frame-to-frame assignment matrix is the one minimizing 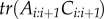. This is a well-known assignment problem, which can be solved by the Hungarian algorithm in 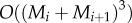 steps, where *M*_*i*_ is the number of detections in frame *i* [27].

#### 2.2.2. Neighbourhood-preserving motion

Actually, when cells are clumped together, they tend to move together, so that the motion of a cell is often correlated with that of its neighbours. To relax the simplifying assumption of independent cell motion, we introduce a heuristic procedure detailed in the electronic supplementary material which repeatedly applies the Hungarian algorithm, each time adjusting the cost matrix in such a way that costs of assignments leading to neighbourhood-preserving motions are lowered. A similar idea was used in [23] to track flows of cell crowds.

### 2.3. Graphical user interface and automated tool calibration

No S&T algorithm can give perfect results when applied to real datasets, errors will likely propagate through S&T iterations. The need for GUIs that easily enable the detection and manual correction of errors is now well recognized [28]. A unique feature of *CellStar* is the ability to manually curate segmentation and tracking results using a GUI and use this information as *ground truth* for parameter learning to further improve batch image processing.

**Figure 4.**
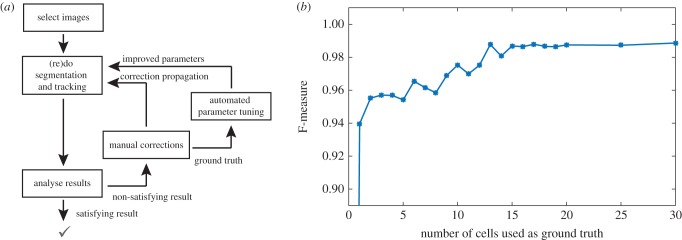
Iterative improvements of S&T performances. (*a*) The GUI facilitates the visualization and correction of mistakes. Manual corrections are used as ground truth and this information is propagated to solve other mistakes. Additionally, they can also serve to learn parameters. (*b*) Typical increase of segmentation quality (F-measure) as a function of the number of cells added to segmentation ground truth. The graph was obtained by applying *CellStar* segmentation to TS3 with some random initial parameters, then repeatedly applying parameter optimization after adding to the ground truth one badly segmented cell at a time. Each time, parameter optimization was not allowed for more than 2 h. (Online version in colour.)

Segmentation ground truth consists of segments drawn, validated or corrected by hand. Thanks to the iterative nature of the segmentation algorithm, manual corrections may propagate and solve other segmentation and tracking issues (figure 4*a*). These contours can be then used for automatic learning of the weights appearing in the definition of the surface energy *E*_surface_. Such weights represent the most delicate segmentation parameters and their tuning by hand is time-consuming. The effectiveness of parameter learning is represented in figure 4*b* on an example taken from our test set. Importantly, learning here amounts to calibrating a few parameters used by the algorithm. It does not require the massive amounts of data needed by machine learning approaches (e.g. deep learning).

Tracking ground truth allows the user to validate or correct the assignment of cells between consecutive frames. Thanks to the iterative heuristic based on neighbourhood-preserving motion, a single correction can propagate to all the neighbour cells, so that a whole cluster of tracking errors may be corrected with a single fix.

Lastly, we implemented a problem-finding procedure that highlights suspicious situations to the user, as for example the sudden appearance or disappearance of cells. The detailed description of GUI features is available in the *CellStar* user manual.

### 2.4. Benchmarks and evaluation platform

**Figure 5.**
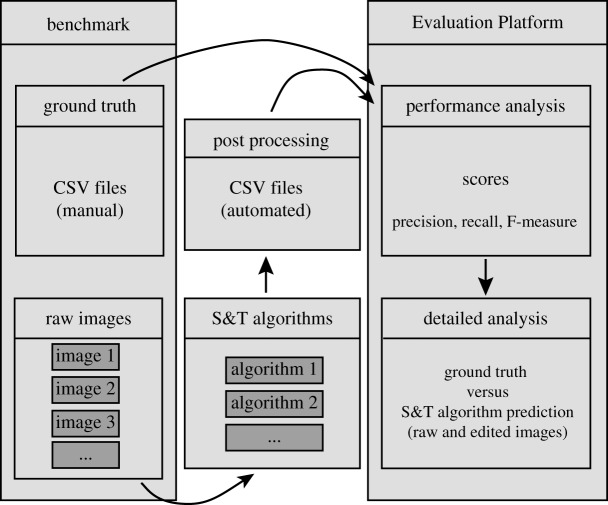
Evaluation Platform (EP) overview. EP requires a benchmark to work, which provides an annotated dataset(s) consisting of raw images and ground truth. Raw images are analysed by an algorithm under investigation, and the outputs of analysis, stored in similar format as ground truth annotations. EP computes precision, recall and *F* scores for all datasets on three problems: segmentation, tracking and long-term tracking. EP outputs contain numerical values summarizing the performance over all datasets, plots providing insights into performance over series of frames as well as annotated images allowing investigation of the performance within the frame (more details in electronic supplementary material, figure S4).

The choice of an image-processing tool is often difficult, because many tools have been proposed so far and because it is impractical to evaluate the effectiveness of each of them by visual inspection, in particular in the case of long-term observations where a considerable amount of data should be inspected by eye. To help with this issue, we created a tool called *Evaluation Platform*. The tool enables convenient evaluation and comparison of the results of algorithms for the segmentation, tracking and long-term tracking of cells (figure 5). The evaluation is based on *benchmark* datasets, consisting of manually annotated images. Given analysis results, the tool computes the percentage of correct elements in the results (precision) and in the ground truth (recall), and computes the F-measure, combining precision and recall. For segmentation, two cells—one in the ground truth and one in the segmentation result—are correctly mapped if their centres are closer than a given threshold distance and they are the closest to each other in both images. Note that we do not assess the correctness of the segmentation at the pixel level. Therefore, our method will detect frequently encountered oversegmentation issues (several predicted cells in a true cell) and undersegmentation issues (one predicted cell spanning several true cells), but may miss small local segmentation issues. In tracking results, links correspond to the positions of a cell centre in two successive frames. Links are correct if they associate the same cell in the tracking ground truth. Lastly, for long-term tracking, the scores are computed identically but using only the first and last image of a movie.

We created an annotated benchmark composed of seven test sets that cover common situations for budding yeast micro-colonies growing as a mono layer, in particular images with sparse cells, dense cells, dividing cells and cells motion (for details, see Materials and methods). It contains more than 12 000 cells to segment and cell pairs to identify and spans up to 3 h of observations (60 frames). Using the *Evaluation Platform*, we compared the performance of seven software tools: Cell Tracer [6], CellID [1], IBSOBT via CellProfiler [25], Tracker [24], CellSerpent [7], CellX [12] and *CellStar*. The tools are listed in electronic supplementary material, table S1. For each algorithm, we dedicated 6 h to tune the tool parameters to the benchmark. During this time, parameters were changed to improve the results, the quality of which was assessed by visual inspection. This approach mimics the typical parameter tuning made by users of imaging software tools. In the case of *CellStar*, we used a combination of manual and automated parameter searches. For the other tools, because automated parameter search strategies were not available, parameter tuning was necessarily manual.

The results of the tools on the benchmark are summarized in table 1. In all tests, *CellStar* obtained the best results for segmentation accuracy, tracking quality and long-term tracking quality. The other software performed well, but none of them was able to obtain consistent results on all test sets for segmentation and tracking. It is worth remarking, however, that the results of the comparison should be interpreted with care, because the results of the tested tools may be different with other parametrizations of the tools, and because not all tracking tools have been included in our test. Camera resolution and temporal frequency of images could also impact results.

**Table 1.** Table summarizes segmentation, tracking and long-term quality (F-measure) in all test sets (green, best; blue, second best). Values were obtained using *Evaluation Platform*. Note that scores for tracking and long-term tracking are not computed on the same set of cells. Tracking quality is computed based on all cells present in the device, whereas long-term tracking quality is computed only based on cells present during the whole experiment. Therefore, tracking quality can be worse than long-term tracking, as observed in some datasets. For CellSerpent, preprocessing was applied to ease background and edge detection and prevent seeds from being between cells. (Online version in colour.)

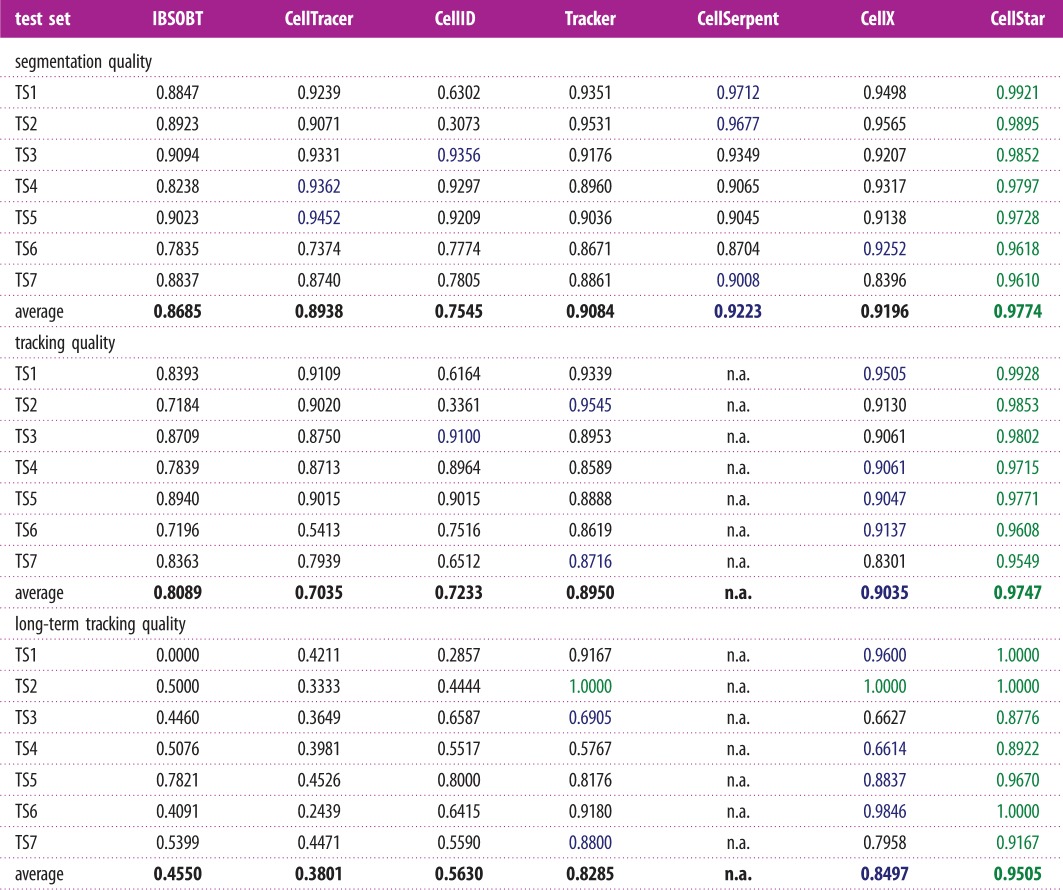

As a community effort, we created the *Yeast Image Toolkit* website from which the *Evaluation Platform* and existing benchmarks can be downloaded. Additional results from other tools and additional manually annotated benchmarks can be freely added by other researchers in the field.

## 3. Discussion

We described *CellStar*, a novel open-source tool to robustly segment yeast cells using brightfield images and to track their trajectories through time with excellent accuracy, which makes this image-processing tool suitable for long-term tracking. The key features of *CellStar* are iterative procedures for segmentation and tracking that exploit additional information to identify and trace cells, such as the cell interior for the identification of cell contours, and cell size, shape and neighbours relative distances for robust cell tracking. *CellStar* comes with a GUI that allows the manual correction of segmentation and tracking errors through easy point-and-click procedures. A few manual interventions are often sufficient to fix all the errors, because most corrections are automatically propagated to neighbour cells by *CellStar* iterative segmentation and tracking procedures. Moreover, manual corrections can be exploited for the automatic learning of the most important parameters, to relieve the user of having to understand all the technical details of the *CellStar* image-processing algorithms. We showed that providing a few correctly segmented cells as ground truth for our learning algorithm enabled it to tune our model and achieve good performance even when starting from default parameter values. The proposed approach should not be confused with machine-learning-oriented approaches that aim to learn a complete model and generally require significant amounts of data [29].

To evaluate the performance of *CellStar* and compare it with other image-processing tools commonly used for yeast, we prepared a benchmark composed of seven tests representative of the most common circumstances occurring in brightfield video microscopy of yeast, including sparse cells, dense cells, dividing cells and cells motion.

We compared *CellStar* with six other software solutions: CellID [1], IBSOBT via CellProfiler [25], CellTracer [6], Tracker [24], CellX [12] and CellSerpent [7]. In all tests, *CellStar* obtained the best results for segmentation, tracking and long-term tracking accuracy. These result should be treated with caution, because every algorithm that we evaluated required parametrization and we cannot guarantee that optimal parameters were found in all cases.

In order to automate the comparison of different tools and have an objective measure of image-processing quality for segmentation, tracking and long-term tracking, we built *Evaluation Platform*, which allows the automatic evaluation and comparison of any segmentation and tracking tool, thanks to benchmarks based on human-supplied ground truth data.

We further worked on the implementation of *CellStar* on different, open-source platforms such as CellProfiler to make it available to a broader community. In contrast to the majority of image analysis tools, the aim here is not for generality regarding the analysis of different cell types (one-size-fits-all approaches). We tailored our approach to a specific, yet extensively studied model organism. We investigate the possibilities to adapt *CellStar* algorithms to other model organisms, such as *Escherichia coli* and *Schizosaccharomyces pombe* (rod shape) without sacrificing performance.

The benchmark we proposed allows the comparison of different existing solutions on the same ground. Together with *Evaluation Platform* and its companion website, it facilitates the exchange of information within the bioimaging community. By resorting to crowdsourcing, we hope to improve the existing tools, collectively develop proper documentation and usage scenarios, collect more data and improve benchmarks. Our initiative is therefore complementary with cell tracking competitions recently initiated [30]. We believe that these two approaches will help biologists to get more reliable quantitative data on the behaviours of individual cells over extended durations. This information is essential to quantify cell-to-cell differences, together with their temporal evolution [31, 32].

## 4. Material and methods

### 4.1. Tools implementations

*CellStar* is implemented in Matlab and is freely downloadable with its documentation at http://www.cellstar-algorithm.org/. The *Evaluation Platform* is implemented in Python and is freely downloadable with its documentation on the Yeast Evaluation Toolkit website at http://yeast-image-toolkit.biosim.eu.

### 4.2. Benchmark construction

We imaged a pSTL1-yECitrine-HIS5, Hog1-mCherry-hph *S. cerevisiae* strain derived from the S288C background [24]. Yeast cells were placed in a microfluidic chamber limiting the growth of the colony to monolayers. We used an automated inverted microscope (IX81; Olympus) equipped with a 100× oil immersion objective (PlanApo 1.4 NA; Olympus) and a QuantEM 512 SC camera (Roper Scientific). The resolution of the camera is 512 × 512. Brightfield images were taken every 3 min (50 ms exposure time).

The benchmark consists of seven test sets that have been extracted from two different acquisitions. They cover a variety of situations, such as isolated cells and small colonies (TS1, TS2 and TS6), colony translations and merging (TS3), big colonies with heavily clustered cells (TS4, TS5 and TS7). For each test set, segmentation and tracking ground truth was prepared in a manual manner by one of the authors and then verified and corrected by another author. The resulting ground truth includes cell centre locations, unique cell number throughout the TS and ‘facultative’ tag. Facultative tags are used to mark the cells on the edge of images (some algorithms discard them by design) and objects that we find questionable. Algorithms are not penalized nor rewarded for discovering or omitting the cells marked as facultative. In total, our benchmark contains more than 12 800 cell segments to process and more than 12 200 cell pairs to identify. More details are in electronic supplementary material, table S1.

### 4.3. Performance evaluation

We computed and compared the quality of segmentation, tracking and long-term tracking of seven different tools. Each tool required some manual parametrization. Because an exhaustive search was not possible, we fixed a maximal amount of time (6 h) to be spent on manual, or in the case of *CellStar*, manual and automated parameter search for each software. From one to three images per test set have been used to tune parameters. Although the resulting parameters depend on the user and its knowledge of the parametrizations used in the image-processing algorithms, this approach mimics the typical usage of the tools and results should reflect both the quality of the underlying algorithms as well as their usability [33].

The quality of the analysis is evaluated using standard criteria. Let *G* be the set of elements in the ground truth, *R* be the set of elements in the algorithm results and *C* the set of associated elements between *G* and *R*. Elements are cells for segmentation evaluations and pairs of cells in successive frames for tracking evaluation. Precision, recall, and *F*-measure are quality criteria defined as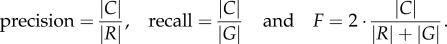 The F-measure combines *precision* and *recall* and represents how similar the result is to the ground truth. In order to improve the fairness and reliability of the F-measure, we added the possibility to specify *facultative elements* in the ground truth, that is inconclusive objects in the images for which the algorithms should not be penalized nor rewarded, neither for finding nor missing them. Let *G*_f_ be the set of cells in the ground truth marked as facultative, 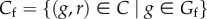. *Precision* and *recall* can then be properly adjusted as 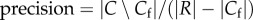 and 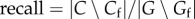.

For segmentation, two cells *r* and *g* correspond to one another (i.e. are in *C*) if *g* is the closest cell in the ground truth and *r* is the closest cell in the results, provided that the distance between them is small.

### 4.4. Automated parameter search in *CellStar*

Parameter learning is currently implemented for segmentation only. Contour and ranking parameters are optimized separately. A contour match measure is used for both optimizations. Given a ground truth contour *θ*, we define the contour match measure of a contour *γ* with respect to *θ* as 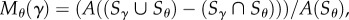 where *A*(*S*) denotes the area of a given surface *S*.

For contour parameter optimization, a few seeds are randomly chosen not far from the centroids of every ground truth contour, and contours are grown from every seed by using the given set of contour parameter values. The ‘cost’ of the parameter set is then defined as the root mean square of all contour match measures. For ranking parameter optimization, several contours are grown from a high number of seeds randomly chosen inside and around every ground truth contours. For each ground truth contour, the contour with the highest rank is selected. The ‘cost’ of the parameter set is then defined as the root mean square of the match measures of all the selected contours.

In both cases, the costs can be minimized using simulated annealing or global search (*simulannealbnd* and *globalsearch* in Matlab). Computation times may vary considerably from minutes to days, depending on the number of ground truth cells, the shape of the cells and the features of the images (contrast, lighting, noise, etc). In our experience on the test set images, good results are obtained within a few hours starting from a random parameter set, and a few minutes are enough for incrementally improving a parameter set after the addition of some new contours to the ground truth.

## Supplementary Material

Supplementary Material

## Data accessibility

The datasets used to benchmark the seven image analysis tools are can be freely downloaded from the YIT website at http://yeast-image-toolkit.biosim.eu.
