# Supplementary material for: Long-term tracking of budding yeast cells in brightfield microscopy: CellStar and the Evaluation Platform
Source: J R Soc Interface. 2017 Feb;14(127):20160705. doi: 10.1098/rsif.2016.0705 (PMC5332563; doi:10.1098/rsif.2016.0705)
Supplement: Supplementary Material [file rsif20160705supp1.pdf]

Long-Term Tracking of Budding Yeast Cells in Brightfield Microscopy: CellStar and the Evaluation Platform -Supplementary Materials-

Cristian Versari [1,†,*], Szymon Stoma [2,†], Kirill Batmanov [1,†,§], Artémis Llamosi [3,4], Filip Mroz [5], Adam Kaczmarek [5], Matt Deyell[3], Cédric Lhoussaine[1,‡], Pascal Hersen[3,‡], Gregory Batt[4,‡]

[1]BioComputing, CRIStAL, Université Lille 1, Lille, France, [2]Scientific Center for Optical and Electron Microscopy (ScopeM), ETH Zurich, Zurich, Switzerland, [3]Laboratoire Matières et Systèmes Complexes, UMR7057, CNRS and Université Paris Diderot, Paris, France, [4]Inria and Université Paris-Saclay, Palaiseau France, [5]Institute of Computer Science, University of Wroclaw, Wroclaw, Poland.

1 Segmentation

Definition of active contours

Active contours (or snakes) are a well-known framework for the identification of object outlines. We refer the reader to [7] for a detailed introduction to active contours. Here, we will focus on the formalization of closed active contours used specifically for the detection of cells. We can then represent an arbitrary contour by means of a "standardized" parameterization $\gamma : [0, l] \to \mathbb{R}^2$ such that:

- γ is continuous, closed (i.e. $\gamma(0) = \gamma(l)$) and bijective (everywhere except in 0 and l), that is a Jordan curve;
- l is the arc length of the curve;
- $|\gamma'(t)|$ is constant and equal to 1 (i.e. γ is the *unit speed* parameterization of the curve);

Contour deformation can be then formalized as the energy-minimization problem

$$\arg\min_{\gamma} : E_{\text{snake}}(\gamma) \text{ with } E_{\text{snake}}(\gamma) = E_{\text{image}}(\gamma) + E_{\text{shape}}(\gamma) + E_{\text{surface}}(S_\gamma)$$

with S_γ being the set of points representing the interior of γ (as defined by the Jordan curve theorem, i.e. the part of $\mathbb{R}^2$ outlined by γ). E_{image} represents an energy term related to the intensity, the gradient and terminations of the image underlying γ. It is the line integral

$$E_{\text{image}}(\gamma) = \int_0^l E_{\text{image}}(\gamma(t))\, \mathrm{d}t = \int_0^l E_{\text{intensity}}(\gamma(t)) + E_{\text{edge}}(\gamma(t)) + E_{\text{termin}}(\gamma(l))\, \mathrm{d}t.$$

* To whom correspondence should be addressed. Contact: Cristian.Versari@univ-lille1.fr

†, ‡ Denotes equal contributions.

§ Current address: Pathology Department, Oslo Univ. Hosp., Norway.

If the snake lies on very bright pixels, $E_{\text{intensity}}$ is correspondingly lower. The same holds for E_{edge} if the snake follows a marked edge, and for E_{termin} if the snake touches visible corners on the image (such as isolated dots, acute angles, or end points of bright edges).

E_{shape} is the *internal* energy of the snake, which depends only on its geometrical properties.

$$E_{\text{shape}}(\gamma) = \alpha\, T(A(S_\gamma)) + \int_0^l \beta\, |\gamma''(t)|\, \mathrm{d}t.$$

where $A(S_\gamma)$ represents the surface area of the interior of γ. The first term accounts for the extent of the snake and the second term measures the regularity of the contour. $T : \mathbb{R} \to \mathbb{R}$ is an arbitrary (continuous) function which allows the introduction of a bias in the energy term depending on the area of the snake, for example to favor the detection of cells with some expected average size.

E_{surface} is the term that makes our approach lie outside of the conventional framework of active contours. The reason why one would avoid such a term (and also the computation of the area $A(S_\gamma)$ in the E_{shape} term) is the high cost of optimizing information coming from a 2D space instead of 1D as in the classical active contour framework. However, the following hypothesis allows us to reduce the cost of such a computation so that the overall computational complexity of the optimization process is not increased. We assume that for every pair of sets $A, B \subset \mathbb{R}^2$ with $A \cap B = \emptyset$ we have $E_{\text{surface}}(A \cup B) = E_{\text{surface}}(A) + E_{\text{surface}}(B)$ (or, more in general, that $E_{\text{surface}}(A \cup B) = \mathbb{F}\big(E_{\text{surface}}(A), E_{\text{surface}}(B)\big)$ for some fixed function $\mathbb{F}$). This allows us to simplify to a great extent the calculation of E_{surface}, making it a double integral of some filter of the image I:

$$E_{\text{surface}}(S_\gamma) = \iint\limits_{S_\gamma} w_1 F_1(x, y) + ... + w_n F_n(x, y)\, \mathrm{d}x\, \mathrm{d}y.$$

$F_1, \ldots, F_n$ are arbitrary image filters that can be easily computed in advance once for all, then integrated over the part of the plane enclosed by the initial contour before starting its optimization. While in general our pipeline handles seamlessly any such filter, in the next section we will describe those specifically used for yeast segmentation.

Overview of *CellStar* segmentation pipeline

We provide an overview of the segmentation pipeline, structured according to the above formalization of E_{snake}. The pipeline consists of the following main steps:

- *preprocessing*, where several filters are applied to the original image: these filters include those used for the calculation of E_{surface} as well as some additional filters needed to place the initial contours;
- *seeding*, where the initial contours (represented by points on the Cartesian plane, in the simplest case) are placed on the image by means of some heuristic search of the points which are supposedly closer to the centroids of cells;
- *contour deformation*, which consists in finding (an approximation of) the "best" contour around each seed according to the costs specified as parameters of the energy function;
- *contour ranking and filtering*, which sorts contours according to the calculated energy function and discards overlapping contours by keeping the ones with the best rank (i.e. lowest energy), similarly to what is done in [2];

The pipeline works in an iterative manner, in the sense that (the last three of) these steps are inserted in a loop. This reflects the fact that the image filters used in the seeding phase can be improved iteration after iteration by including information about the already identified cells. Thanks to this expedient, the final result is considerably improved by concentrating the computational efforts on the regions of the image where cells are to be expected but have not been found yet.

```
/* Preprocessing */
 · compute background image (generally once for every movie, computed over
     one or more images of the movie)
 · compute foreground_mask, clean_image, cell_content and cell_border images
     from current image

 for step = 1 to max_seeding_steps do
     /* Seeding */
     if (step == 1) then
         · collect seeds from (unmodified) cell_content and cell_border images
     else
         · add to seeds the centroids of current segments
         · add to seeds new points randomly chosen around existing seeds
         · modify cell_content and cell_border images by adding current segments
         · collect seeds from modified cell_content and cell_border images
     end if
     · remove duplicates in seeds according to a given min_distance threshold
     if no new seeds have been collected then
         break loop
     end if

     for each new seed in seeds do
         /* Initialization of polar contour */
         · compute radii originating from current seed
         · compute Ẽ_snake = local approximation of E_snake along each of the radii
         · identify minima of Ẽ_snake along each of the radii
         · compute initial contour by connecting coordinates of minima
         /* Contour deformation */
         while contour regularity constraint is not satisfied do
             · pick the farther point from seed where the contour is not regular
             · identify new_minimum of Ẽ_snake along the radius corresponding
                 to such a point so that the regularity constraint is locally satisfied
             · replace the original point in contour with the one
                 corresponding to the new_minimum
         end while

         /* Contour ranking */
         · compute (better approximation of) E_snake for the obtained contour
         · add contour to the current set of contours
     end for

     /* Contour filtering and creation of segments */
     · remove "bad" contours (too big, too small, too high E_snake, etc.)
     · sort all contours by increasing E_snake
     · discard all previously computed segments in the current image
     for each contour in sorted contours do
         if current contour interior overlaps with existing segments more
             than a given max_allowed_overlapping threshold then
             · discard contour
         else
             · create new segment from non assigned pixels in the interior of
                 current contour
         end if
     end for
 end for
```

Figure S1. Sketch of segmentation pipeline in pseudocode. The main sates of the pipeline (preprocessing, seeding, contour deformation, ranking and filtering) are indicated by the corresponding remarks between `/*` `*/`.

The pipeline is graphically sketched in Figures 2 and 3, while a complete description in pseudo-code is given in Figure S1. We proceed now with the detailed description of each step.

Preprocessing

The aim of the preprocessing part is the application of a series of filters to the original image to compute a set of intermediate images representing all the features exploited later on in the proper segmentation phase. They are obtained by applying some classic filters for the attenuation of noise and illumination artifacts, edge detection, as well as several custom filters for the computation of binary masks necessary for efficient seeding and contour deformation. We mention in particular the following intermediate images obtained from the original light microscopy image(s), for each of which an example is shown in Figure 2.

Background image. The background image can be thought as the picture that would be taken by a microscope without any cell in the field of view. Under optimal conditions, this picture should be totally "empty", that is its gradient should be exactly zero everywhere, or in other words it should look as a perfectly uniform gray field. In reality, this is often not the case for several reasons: imperfections on the surface of the microfluidic device, foreign objects above or below the specimen, non optimal settings of the microscope causing lighting artifacts, just to cite some possible causes.

A non uniform background may heavily affect the result of the segmentation process in a negative way, especially if the imperfections overlap cells. For this reason, segmentation quality can be improved by subtracting the background from the original image (thus obtaining the *clean image*, described later). If a background image is not available, an approximation of it can be computed in several ways. The *CellStar* tool includes many filters that can be arbitrarily combined to this purpose, taking advantage of all the available information including human knowledge, if available, through an interactive user interface. For more details we refer to *CellStar* user manual.

Clean image. The clean image is obtained by subtracting the background from the original image, under the hypothesis that

- lighting conditions are the same in the background and in the original image (otherwise filters must be applied to adjust contrast and/or brightness);
- both the elements in the background (if any) and the cells to be segmented are partially transparent, and their superimposition is additive in terms of absorbed light (or phase shift, if the images are obtained by phase contrast).

The clean image coincides (up to uniform shift) with the original image if the background is uniform. Since the background is rarely capturing all the extraneous elements, the clean image is modified in a later step by the application of the foreground mask, described in the following.

Foreground mask. The foreground mask is a binary image indicating the points of the surface where cells are supposedly located. If the background image captures all the foreign elements and there is no noise, the foreground mask can be easily computed by selecting the points where the background and the original image differ, i.e. where the clean image is not empty. However this is not the case in practice, because noise is always present in image capture and, more importantly, in most of the cases the available background image represents just an approximation of the real background. Therefore, a series of operations are performed by *CellStar* to define the foreground mask.

In detail, we first select the set of pixels where the background image and the clean image differ by more than a given threshold. This gives a first approximation of the foreground image. However, some regions inside cells and along cell borders are missing. A small dilation operation of the obtained mask solves a number of such problems. If there remain non-selected (i.e. black) regions that are smaller than the smallest admissible size of a cell within the current foreground

mask, we iterate a sequence of (i) integrating these regions in the current mask, and (ii) eroding and dilating the current mask, always by the same small distance, until no black region smaller than a cell size remain within the foreground mask. A final step consists in removing all the selected (i.e. white) regions that are smaller than the smallest admissible cell size.

Cell content and cell border images. Depending on the optical microscopy technique used, images highlight in particular ways the structural details of the specimen. It can be useful for segmentation to split the clean image into two complementary images: the *bright image*, which includes the pixels with high brightness (more precisely, it is equal to the clean image where the original image is brighter than the background and the foreground mask is white, while it is flat everywhere else), and the *dark image*, defined in a symmetric way. The application of thresholds to these images produces respectively the *bright* and *dark masks*.

In *CellStar*, these images are exploited in several steps of the tool chain, in particular during the seeding phase to compute the starting points of the snakes to be settled. In the case of phase contrast microscopy images, cell borders and interior are well represented by either the bright or the dark image (and masks). For this reason, in *CellStar* implementation we chose to conventionally name the bright image *cell border image*, and the dark image *cell content image* (and the same for their respective masks) assuming positive phase contrast imaging of yeast cells. Negative phase contrast images can of course be easily treated by segmenting the negatives of the images. An example of cell content mask is shown in Figure 2.

Seeding

In many implementations of active contours used for the automatic segmentation of cell images, the seeding phase is of fundamental importance for the identification of all the cells. Alternative approaches exist where the seeding phase is not necessary (see [9] for an interesting example), but their adaptation to the difficult conditions present in cell imaging seems non trivial.

Seeds represent the starting solution for the optimization of snakes by energy minimization, whose final contour lies usually not far from the seed itself. For this reason, if no seed is placed in the surroundings of a cell during the seeding phase the cell will most likely be missed at the end of the segmentation process. To avoid this issue, it would be of course possible to place seeds nearly everywhere on the image but this would lead to two different problems. First, the overall computational efficiency would be heavily affected (in particular for non crowded images) because of the very high number of seeds to be treated in every image. Second, a clever post-processing phase would be needed to discard all the "false positives" (the contours not corresponding to an existing cell) as well as to resolve all the conflicts caused by overlapping snakes. While such a post-processing phase is actually included in *CellStar* pipeline anyway (see the section *Contour ranking and filtering*), smarter seeding strategies are also implemented to keep a good computational efficiency as well as to provide the widest range of compromises between speed and quality.

Some of these strategies are specific to the parameterization of active contours currently implemented in *CellStar* pipeline for efficiency reasons (see section *Contour deformation* for details), which is quite sensitive to the position of seeds, simply represented by a point on the image: it requires in particular that the seed lies *inside* the final outline of the snake, and the best precision in terms of cell shape approximation is obtained when the seed is reasonably close to the centroid of the cell.

While *CellStar* pipeline may seamlessly integrate any seeding strategy (as well as many other parameterizations of active contours), the current implementation includes seeding from:

- the cell border image: the centroids of cells are roughly approached by looking for brightness minima after applying smoothing filters (similarly to the seeding strategy implemented in [2]);
- the cell content image: the previous strategy can be replicated by considering the maxima of the cell content image;

- random points around the seeds described in the previous categories: this technique allows overcoming local noise that sometimes prevents the proper expansion of the snake when using local energy minimization strategies (see section *Contour deformation*).

Seeding from the cell border image gives often the best results at the beginning, while the other strategies are useful in the subsequent steps of the main iteration loop.

Moreover, the repetition of the segmentation phases allows the introduction of two further important strategies (to which randomization is also applied as described in the last of the previous strategies):

- computation of seeds from the centroids of existing segments: using previous segments centroids often allows the computation of seeds closer to the real centroid of the cell, leading in the end to a more faithful tracing of cell outline, by exploiting the same idea explained in [4] and [1] (see Figure 3A for more details);

- seeding from modified cell border and content images (see Figure 2): the exclusion of existing segments from the cell border and content images leads to the identification of new minima and maxima, often allowing the discovery of new cells in a very efficient way.

To further increase efficiency, *CellStar* includes seed filtering to remove "duplicate" seeds (based on a configurable threshold distance).

The main iteration loop is interrupted when no new seeds are found, or when the maximal number of desired repetitions has been reached.

Contour deformation

Contour deformation is the most critical phase of the segmentation process in terms of computational efficiency, because of the high freedom of the active contour parameterization γ which allows the snake to take any non self-intersecting closed shape. While our segmentation pipeline may exploit (even at the same time) several different parameterizations of active contours and energy minimization strategies among the existing ones (polar or generic, global or local, such as [1, 2, 8]), for efficiency reasons we chose to provide a new ad hoc local minimization algorithm exploiting one of the most efficient parameterizations proposed so far for closed active contours: *polar active contours* (or *active rays*, introduced in [4]).

Polar active contours allow removing one dimension in the minimization of the snake energy function, passing from 2D optimization to 1D, at the price of reducing the freedom of the contour parameterization γ to a subset of Jordan curves: those corresponding to outlines of star-shaped sets, referred to as *stars* hereafter.

Formally, given a point (x_m, y_m), a star is uniquely represented by a 1D function $\rho : [0, 2\pi] \to \mathbb{R}^+$ such that for every angle $\phi \in [0, 2\pi]$, a point $\gamma(t)$ of the contour of the star can be described by

$$\gamma(t) = \gamma(\sigma(\phi)) = (x_m + \rho(\phi)\cos(\phi), y_m + \rho(\phi)\sin(\phi))$$

for some proper bijective function $\sigma : [0, 2\pi] \to [0, 1]$ that allows mapping angles to the standardized parameterization of γ previously described (reason why we also need to impose $\rho(0) = \rho(2\pi)$).

Not for all Jordan curves it is possible to find a point (x_m, y_m) and a function ρ to obtain a description in polar coordinates. This limitation is important for its practical implications. The most critical implication is that the typical shapes of some kinds of cells cannot be properly captured by this polar parameterization, although many others can. A second minor drawback is that the segmentation of a cell can be accomplished only if the point (x_m, y_m) lies in the interior of the cell being segmented, and possibly not too close to its border. This issue is however easily overcome by exploiting a proper seeding strategy as described in the previous section.

On the other hand, the advantages of such a parameterization are remarkable in terms of computational efficiency and ease of implementation. In particular, it allowed us to provide an ad-hoc implementation of the minimization of E_{snake} that meet the most demanding constraints in

terms of time (as in the case of real-time image processing) and segmentation quality (needed for long-term cell tracking). To achieve this result, we approximated the original objective function to be minimized through a series of discretization procedures and split the global optimization problem into a sequence of local minimization steps, each one considering only a subset of the parameters to be optimized.

The roughness of the discretization is correlated to the speed of the segmentation (as well as inversely correlated to the quality of the result), and can be tuned through *CellStar* user interface. Among other parameters, the discretization applies to:

- the domain of the function ρ, considered only on a subset of r equispaced points in the interval $[0, 2\pi]$, corresponding to taking into account only r equispaced rays originating from (x_m, y_m);
- the codomain of the function ρ, allowed to assume values only in a subset of n equispaced points in $]0, N_{\max}]$ for some $N_{\max}$ specified by the user and representing the maximal allowed radius of a cell;
- the calculation of all the energy terms, that are sampled according to the discretization of ρ.

The approximated minimization algorithm consists of three main steps:

1. a very rough sampling of E_{snake} computed along each ray independently, according to the above discretization and taking the initial seed as the point (x_m, y_m): in Figure 2H, this is shown as a circle with radial sectors of variable brightness, corresponding to the (negative of the) computed approximation of E_{snake} on all the points lying at a distance lower than $N_{\max}$ from the seed;
2. the identification of an extended initial contour, obtained simply by taking the point of minimal energy along each ray (thus temporarily disregarding the energy term related to contour regularity): an example is shown as set of red dots in Figure 2H;
3. the minimization of the computed approximation of E_{snake} by means of a local optimization strategy which shrinks the extended contour, leading for example to the contour colored in blue in Figure 2K.

The energy E_{snake} approximated at the step 1 includes the term E_{surface}, that in turn includes several double integrals computed on the foreground, clean, cell border and cell content images previously described, each one weighted according to a tunable parameter.

CellStar user interface allows also automatic tuning of these and other parameters figuring in the definition of E_{snake} thanks to a supervised optimization algorithm.

Contour ranking and filtering

Contour ranking and filtering is the last phase of segmentation, which takes care of

- computing a more faithful approximation of E_{snake} for each snake: double integrals in particular (including the area of contours) are recalculated by means of a more accurate algorithm optimized for star-shaped segments, and re-weighted by another set of parameters which can also be tuned automatically through supervised learning;
- removing segments that do not satisfy some basic criteria (size, minimal or maximal thresholds on the integrals included in the calculation of E_{surface}, etc.): an example is shown in Figure 3E and F;
- sorting segments according to their energy and resolving segmentation conflicts by removing snakes overlapping with other, lower-energy snakes (similarly to what is done in [2]).

A positive side effect of the resolution of conflicts for overlapping snakes is that this final phase of filtering constitutes a further step in the (global, this time) minimization of the average of the energy E_{snake} among all the segments.

At the end of this phase, segmentation is either restarted from the seeding after that cell border and cell content images have been modified by taking into account the segments freshly found, or it is interrupted if the maximal number of steps has been reached, so to start with tracking.

2 Tracking

We start by describing how segmented cells in two successive frames are related. Then, we show how full cell traces are constructed using this information. Finally, we present an improvement of the algorithm where we relax the assumption that cells move independently of each other.

Frame-to-frame assignments

In the following, we assume that N is the number of frames and M_i is the number of segmented cells in frame number i.

We introduce *frame-to-frame assignment matrices* to relate cells on two successive frames. More precisely, a frame-to-frame assignment matrix $A_{i:i+1}$ is a square matrix of boolean (0 or 1) values that relate lth cell detection on frame i with mth cell detection on frame $i+1$ if they correspond to the same cell identity, and taking into account appearing and vanishing cells between both frames. The general form of this matrix in pictured in Figure S2. Formally,

$$A_{i:i+1} = [a_{lm}^{(i:i+1)}]_{1 \leq l,m \leq M_i + M_{i+1}}$$

is a symmetric matrix where, for $l \leq m$, we have $a_{lm}^{(i:i+1)} = 1$ iff

- $l \leq M_i$, $m > M_i$, and d_{il} and $d_{i+1,m-M_i}$ are detections of the same cell identity, or
- $l = m \leq M_i$ and the cell detected as d_{il} is not present in frame $i+1$, or
- if $l = m > M_i$, then the cell detected as $d_{i+1,m-M_i}$ is not present in frame i.

This defines entirely the matrix $A_{i:i+1}$ by symmetry. Moreover, by construction, $A_{i:i+1}$ has exactly one non-zero value in each row and in each column. This means that each detection is either marked as having a single corresponding detection in another frame, or as having no corresponding detection.

$$\begin{array}{ccc} & & \ldots\ldots m \ldots\ldots \quad \leftarrow \text{detection number of } (i+1)\text{th image} \\ A_{i:i+1} = & \vdots\, l\, \vdots & \left(\begin{array}{c|c} D_1 & A \\ \hline A^T & D_2 \end{array}\right) \\ & \uparrow & \text{detection number of } i\text{th image} \end{array}$$

Figure S2. Frame-to-frame assignment matrices. D_1 and D_2 are diagonal matrices that refer to cells detected resp. up-to image i and from image $i+1$. Matrix A relates detections numbers of the same cell.

Now the task is to find the best frame-to-frame assignment $A^*_{i:i+1}$. Here, we assume that each cell moves (in general, changes) independently of the others. We will weaken this assumption later, but it provides a useful starting point, because it leads directly to an efficient solution.

We will use the (symmetric) cost matrix $C_{i:i+1} = [c_{lm}^{(i:i+1)}]_{1 \leq l,m \leq M_i + M_{i+1}}$, whose entries $c_{lm}^{(i:i+1)}$ are defined as follows (by symmetry, we restrict the definition to the triangle $l \leq m$).

- for $l = m \leq M_i$ and $l = m > M_i$, the costs of "missing cells" assignments, are set to one of two constants, depending on whether the corresponding detection is judged "reliable". A detection is considered reliable if its centroid lies farther than a certain threshold from frame borders and its area is greater than a certain threshold. The reason is that cells near borders tend to appear and disappear more frequently, as they are coming in and out of the field of view, and segmentation of small cells is not as reliable as bigger ones, which also causes frequent disappearance. In addition, small cells can be born at this frame, which naturally makes them appear out of nowhere. Correspondingly, the cost of a reliable detection having a missing counterpart is higher than that of an unreliable detection.

- for $l \leq M_i$ and $m > M_i$, the cost $c_{lm}^{(i:i+1)}$ of associating two detections with a single cell depends on how similar detections d_{il} and $d_{i+1,m-M_i}$ are. In general we can take into account any features of the detections. We consider $d_{ij} = (x_{ij}, s_{ij})$, where x_{ij} are the 2d coordinates of the centroid of the detection and s_{ij} is the area of the cell. These quantities are normalized with respect to expected adult cell size, which is given or computed during preprocessing stage. We define the following expression for the cost:

$$c_{lm}^{(i:i+1)} = \|x_{il} - x_{i+1,m}\| - w_a \frac{\min(s_{il}, s_{i+1,m})}{\max(s_{il}, s_{i+1,m})} \quad (1)$$

  where w_a is a parameter specifying relative importance of preserving the area during matching.

- finally, $c_{lm} = \infty$ for all other values of l and m for $l \leq m$. In practice, one can actually take any value greater than the other costs for ∞.

The best assignment is then the one minimizing the trace of the product $A_{i:i+1}C_{i:i+1}$.

$$A^*_{i:i+1} = \min_{\{A_{i:i+1} | a_{lm}^{(i:i+1)} \in \{0,1\}, \forall q \in \{1 \dots M_i+M_{i+1}\}, \sum_{p=1}^{M_i+M_{i+1}} a_{pq}^{(i:i+1)} = \sum_{p=1}^{M_i+M_{i+1}} a_{qp}^{(i:i+1)} = 1\}} tr(A_{i:i+1}C_{i:i+1})$$

This expression is the formulation of a well-known assignment problem, which can be solved by the Hungarian algorithm in $O((M_i + M_{i+1})^3)$ steps where M_i is the number of detections in frame i [10].

Cell trace reconstruction

A solution of the tracking problem can be represented by a trace matrix

$$T = [t_{ik}]_{1 \leq i \leq N, 1 \leq k \leq K} \quad \text{where} \quad t_{ik} = \begin{cases} j & \text{if in frame } i \text{ cell } k \text{ has detection } d_{ij} \\ 0 & \text{if cell } k \text{ is not present in frame } i. \end{cases}$$

and K is the total number of cells observed during the experiment, which also has to be estimated.

We now describe a procedure for trace matrix reconstruction. It uses the number K_i of detected cells up to frame i. Thus, $K_1 = M_1$ and K_{i+1} is K_i plus the number of new cells detected in frame $i+1$ as specified by $A_{i:i+1}$, that is the number of detection numbers $m > M_i$ such that $a_{mm}^{(i:i+1)} = 1$. T is then defined as $Join(\{A_{i:i+1}\}_{1 \leq i < N})$, where

- in the first frame the kth detection is assigned to cell identity k (i.e. $t_{1k} = k$ for $k \leq M_1$ and $t_{1k} = 0$ otherwise),
- in the frame $i+1$, for $1 \leq i < N$,
  - mth detection is assigned to cell identity $k \leq K_i$ (i.e. $t_{i+1,k} = m$) if this one was assigned lth detection in the previous frame (i.e. $t_{i,k} = l$) and, if m and l are related in the matrix $A_{i:i+1}$ (i.e. $a_{l,m+M_i}^{(i:i+1)} = 1$),
  - mth detection is assigned to cell identity $K_i + k \leq K_{i+1}$ (i.e. $t_{i+1,k+K_i} = m$) if $m + M_i$ is the kth new detected cell in the matrix $A_{i:i+1}$ (i.e. such that $a_{m+M_i,m+M_i}^{(i:i+1)} = 1$),

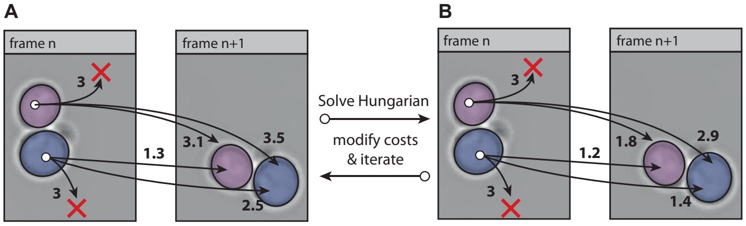

Figure S3. Illustrative example of improvement of tracking quality by neighborhood-preserving motion. **A**: The cost of each possible cell assignment is computed without knowledge of neighbors relocation. By applying the Hungarian algorithm the lower cell in frame n is (incorrectly) assigned to the left cell in frame n+1 while the other cell is (again, incorrectly) marked as disappeared. **B**: the costs of assignments are recomputed by taking into account the neighborhoods computed in **A**. The presence of the cell incorrectly mapped increases the chances of assigning its neighbors in frame n to some of its neighbors in frame n+1. With this new information, assignment costs change so that the overall tracking precision considerably improves.

– 0 is assigned to all other cell identities.

This procedure is entirely determined by the frame-to-frame assignment matrices except for the choice of cell identities attributed to newly detected cells which is arbitrary and given here in the detections' order.

Neighborhood-preserving motion

Our heuristic algorithm proceeds by iterations until convergence. On the initial iteration, the independent motion model is used. This gives an initial approximation of assignments. On the next iteration, when deciding the cost of an assignment c_{lm}, we examine assignments of neighbors of the detection d_{il} in frame i, i.e. detections which are closer than a threshold to d_{il}. If there are no neighbors or no neighbors have been assigned to a detection in frame $i+1$, the cost is computed as before. If there are assigned neighbors, instead of using the distance in the cost (1) we use $\min_{p|d_{ip} \text{ near } d_{il}} \|(x_{il} - x_{i+1,m}) - (x_{ip} - x_{i+1,q})\|$, where $d_{i+1,q}$ has been assigned to d_{ip} on the previous iteration. The idea behind this expression is that the shift of a neighbor, as computed on the previous iteration, can compensate the cost of a similar shift for the cell under consideration. Only the difference between shifts is added to the cost. By taking the minimum cost among all neighbors, any cell shift computed on the previous iteration allows all its neighbors to shift in the same direction with low cost. This means that if there was at least one good assignment, it will spread in a wave-like manner, each iteration computing good assignments for another "layer" of neighbor cells.

In this extended algorithm, it is critically important to have features other than positions on which the probability of an assignment can be based. The most important feature is the cell size, which is the only one we include in the computation. It is easy to first assign a few unusually large cells with high confidence (*i.e.* low cost), and then spread this confidence across the field in several iterations. Relatively big group shifts, with displacements greater than a cell diameter, which happen in many long experiments, can be resolved this way. If the tracking algorithm does not take the neighborhood into account, it will fail massively in such conditions, assigning all cells in such a group wrongly. Therefore this feature greatly improves the robustness of the long-term tracking with respect to such perturbations. Typically the process converges in 2 to 3 iterations, while some harder cases took up to 6 iterations.

Test set<br>Duration	Cells per frame<br>Frame number	Number of cells<br>Number of pairs	First frame	Middle frame	Last frame
**Test Set 1**<br>240 min	from 14 to 26 cells<br>60 frames	1167 cells<br>1154 cell pairs	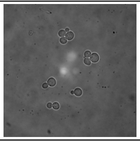	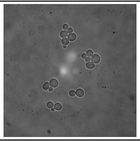	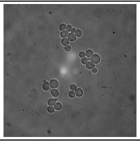
**Test Set 2**<br>90 min	from 4 to 6 cells<br>30 frames	145 cells<br>141 cell pairs	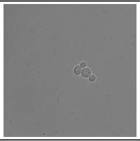	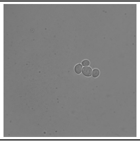	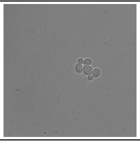
**Test Set 3**<br>60 min	from 101 to 128 cells<br>20 frames	2285 cells<br>2183 cell pairs	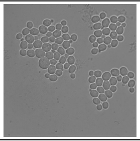	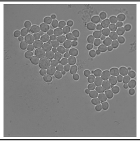	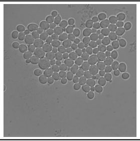
**Test Set 4**<br>60 min	from 169 to 237 cells<br>20 frames	4171 cells<br>4002 cell pairs	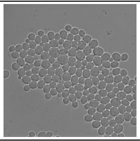	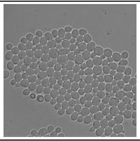	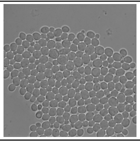
**Test Set 5**<br>60 min	from 143 to 173 cells<br>20 frames	3092 cells<br>2949 cell pairs	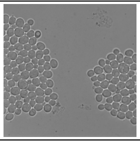	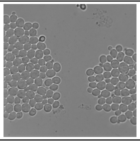	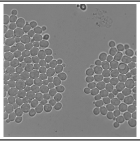
**Test Set 6**<br>40 min	from 36 to 49 cells<br>10 frames	442 cells<br>406 cell pairs	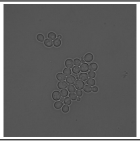	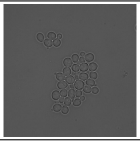	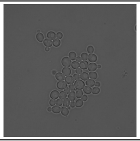
**Test Set 7**<br>40 min	from 129 to 184 cells<br>10 frames	1541 cells<br>1412 cell pairs	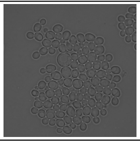	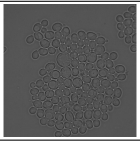	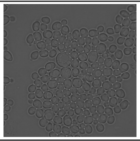

Table S1. Table summarizing test sets used in our benchmark.

3 Benchmarks and evaluation platform

Our benchmark is made of seven different test sets, chosen to cover the diversity of problems typically-encountered in yeast cell imaging. These include: small colonies (TS1, TS2, TS6), heavy cell clustering (TS4, TS5, TS7) and colony merging (TS3) which is especially challenging for any tracking algorithm. For each frame in test sets, we provide the original image, the coordinates of the centers of all cells, and the ID of the corresponding cell in the previous frame. Note that we do not provide full segmentations for cells. Manual segmentation would be extremely time consuming (more than 12800 cells are in the benchmark) and debatable (the delimitation of cell frontiers is often a matter of appreciation). By comparing only the position of cell centers, we may miss local segmentation issues. Yet our method will correctly detect all oversegmentation and undersegmentation problems, that are the most common segmentation issues encountered. The seven test sets are briefly described in Table S1.

The *Evaluation Platform* scores the segmentation and tracking results provided by an algorithm based on the ground truth given as an input (Fig. S4). Because the interpretation of some situations is ambiguous even for the human expert, our tool accounts for the possibility to have facultative

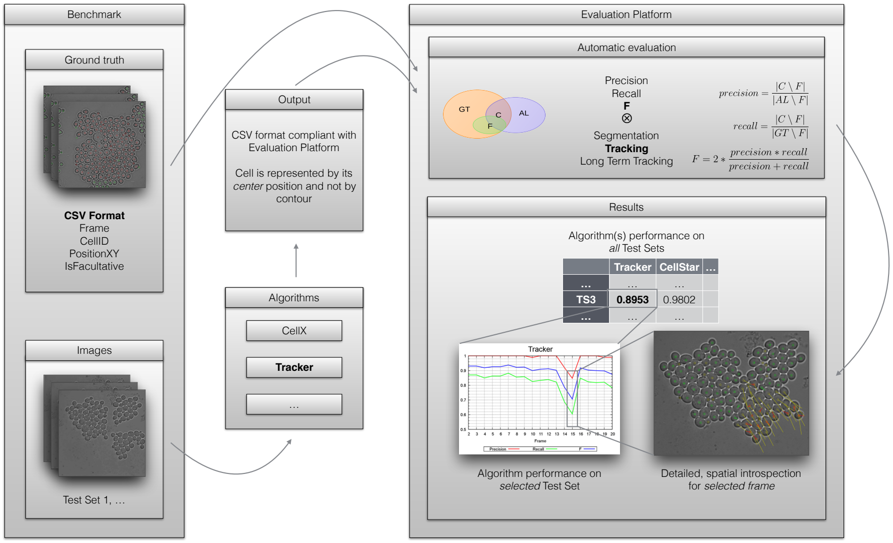

Figure S4. Evaluation Platform (EP) overview. EP requires a benchmark to work, which provides annotated data set(s) consisting of raw images and ground truth. Raw images are analyzed by an algorithm under investigation (e.g. Tracker), and the outputs of analysis must be stored in similar format as ground truth annotation. EP computes precision, recall and F scores for all data sets in three areas: segmentation, tracking and long-term tracking. EP outputs contain numerical values summarizing the performance over all datasets, plots providing insights into performance over series of frames (here F-measures for Tracker on Test Set 3 are shown) as well as annotated images allowing to investigate performance within the frame.

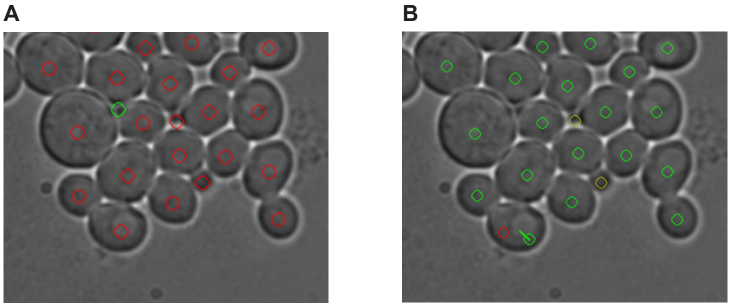

Figure S5. *Evaluation Platform* scores the segmentation provided by an algorithm based on the ground truth (GT) provided as an input. (A) Segmentation ground truth. Annotations are presented as circles. Obligatory cells and facultative objects are marked with red and green circles respectively. (B) Score details for segmentation. Correct matches are marked with green (with lines presenting GT and result disparity), false positives and negatives are marked with red and yellow respectively.

objects in the ground truth. Decisions taken by the algorithms for these facultative objects do not impact the segmentation or tracking score of the algorithms under study (Fig. S5).

The performance of several algorithms has been tested using the evaluation platform on our benchmark data set. The list is provided in Table S2. Note that not all tools are implementing segmentation *and* tracking procedures. The results that we obtained on one test set are provided in details in the Figure S6 and S7, for segmentation and tracking respectively. The mean performance

Program	Software webpage	Publication
IBSOBT (CellProfiler)	http://cellprofiler.org/	[3]
CellID	http://lbms.df.uba.ar/	[6]
Cell Tracer	http://www.stat.duke.edu/research/software/west/celltracer/	[12]
CellSerpent	http://microscopy.uni-graz.at/index.php?item=new2	[2]
CellX	http://www.csb.ethz.ch/tools/software/cellx.html	[5]
Tracker	Upon request	[11]
Cell Star	http://cellstar-algorithm.org	this article

Table S2. Table summarizing programs used for comparison.

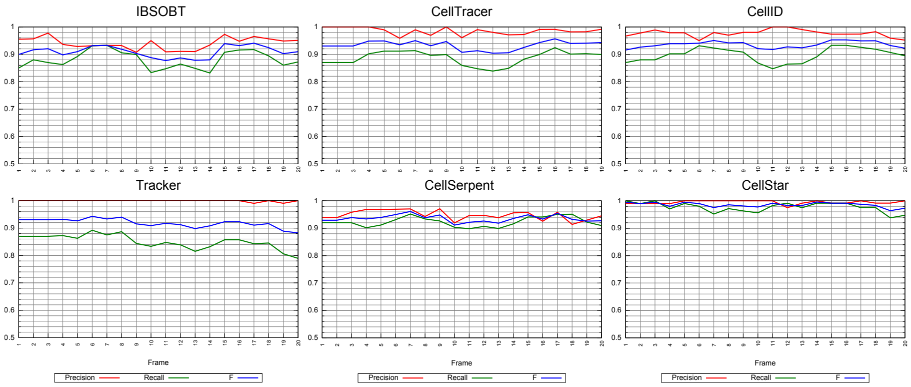

Figure S6. Segmentation evaluation presented as *precision*, *recall* and $F - measure$ plots for test set 3.

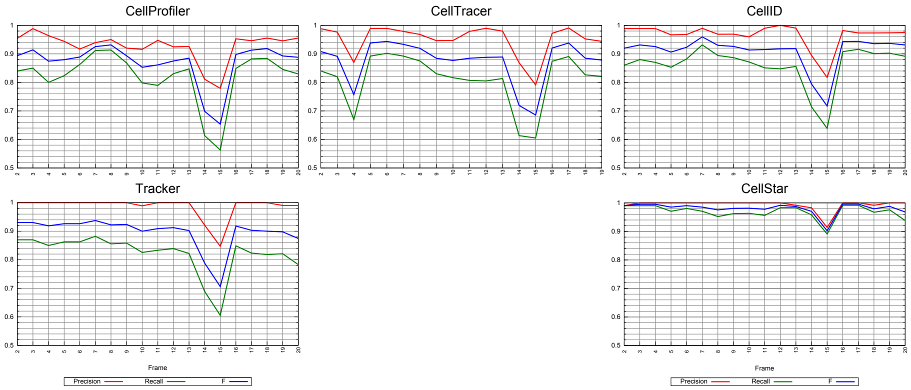

Figure S7. Tracking evaluation presented as *precision*, *recall* and $F - measure$ plots for test set 3. The sudden drop at the frame 15 corresponds to a difficult situation present in test set 3: the merge of colonies. All tested algorithms have problems with correct assignment of cells in this situation.

is provided on Table 1 in the main text. They are also collected on the Yeast Evaluation Toolkit, with the ambition to initiate a larger research effort.

References

[1] P. Bamford and B. Lovell. Unsupervised cell nucleus segmentation with active contours. *Signal Processing*, 71(2):203--213, 1998.

[2] K. Bredies and H. Wolinski. An active-contour based algorithm for the automated segmentation

of dense yeast populations on transmission microscopy images. *Computing and Visualization in Science*, 14(7):341--352, 2011.

[3] A. E. Carpenter, T. R. Jones, M. R. Lamprecht, C. Clarke, I. H. H. Kang, O. Friman, D. A. Guertin, J. H. H. Chang, R. A. Lindquist, J. Moffat, P. Golland, and D. M. Sabatini. CellProfiler: image analysis software for identifying and quantifying cell phenotypes. *Genome Biology*, 7(10):R100, 2006.

[4] J. Denzler and H. Niemann. Active rays: Polar-transformed active contours for real-time contour tracking. *Real-Time Imaging*, 5(3):203--213, 1999.

[5] S. Dimopoulos, C. Mayer, F. Rudolf, and J. Stelling. Accurate cell segmentation in microscopy images using membrane patterns. *Bioinformatics*, 30(18):2644--2651, 2014.

[6] A. Gordon, A. Colman-Lerner, T. E. Chin, K. R. Benjamin, R. C. Yu, and R. Brent. Single-cell quantification of molecules and rates using open-source microscope-based cytometry. *Nature Methods*, 4(2):175--181, 2007.

[7] M. Kass, A. Witkin, and D. Terzopoulos. Snakes: Active contour models. *International journal of computer vision*, 1(4):321--331, 1988.

[8] M. Kvarnström, K. Logg, A. Diez, K. Bodvard, and M. Käll. Image analysis algorithms for cell contour recognition in budding yeast. *Optics Express*, 16(17):12943--12957, 2008.

[9] W. Li, S. Song, and X. Qian. Active contours with selective local or global segmentation property for multiobject image. *Optical Engineering*, 50(6):067009--067009, 2011.

[10] J. Munkres. Algorithms for the assignment and transportation problems. *Journal of SIAM*, 5:32--38, 1957.

[11] J. Uhlendorf, A. Miermont, T. Delaveau, G. Charvin, F. Fages, S. Bottani, G. Batt, and P. Hersen. Long-term model predictive control of gene expression at the population and single-cell levels. *Proceedings of the National Academy of Sciences of the USA*, 109(35):14271--14276, 2012.

[12] Q. Wang, J. Niemi, C.-M. Tan, L. You, and M. West. Image segmentation and dynamic lineage analysis in single-cell fluorescence microscopy. *Cytometry Part A*, 77(1):101--110, 2010.
